# Injury epidemiology in Iran: a systematic review

**DOI:** 10.5249/jivr.v9i1.852

**Published:** 2017-01

**Authors:** Saber Azami-Aghdash, Homayoun Sadeghi-Bazargani, Hosein Shabaninejad, Hassan Abolghasem Gorji

**Affiliations:** ^a^Department of Health Services Management, School of Health Management and Information Sciences, Iran University of Medical Sciences, Tehran, Iran.; ^*b*^Road Traffic Injury Research Center, Tabriz University of Medical Sciences, Tabriz, Iran.; ^*c*^Center of Excellence in Health Management and Economics, Iran University of Medical Sciences. Tehran, Iran.

**Keywords:** Injury, Trauma, Epidemiology, Pattern

## Abstract

**Background:**

Injuries are the second greatest cause of mortality in Iran. Information about the epidemiological pattern of injuries is effective in decision-making. In this regard, the aim of the current study is to elaborate on the epidemiology of injuries in Iran through a systematic review.

**Methods:**

Required data were collected searching the following key words and their Persian equivalents; trauma, injury, accident, epidemiology, prevalence, pattern, etiology, risk factors and Iran. The following databases were searched: Google Scholar, PubMed, Scopus, MagIran, Iranian scientific information database (SID) and Iran Medex. Some of the relevant journals and web sites were searched manually. The lists of references from the selected articles were also investigated. We have also searched the gray literature and consulted some experts.

**Results:**

Out of 2747 retrieved articles, 25 articles were finally included in the review. A total of 3234481 cases have been investigated. Mean (SD) age among these cases was 30 (17.4) years. The males comprised 75.7% of all the patients. Only 31.1% of patients were transferred to hospital by ambulance. The most common mechanism of injuries was road traffic accidents (50.1%) followed by falls (22.3%). In road traffic accidents, motorcyclists have accounted for the majority of victims (45%). Roads were the most common accident scene for the injuries (57.5%). The most common injuries were to the head and neck. (47.3%). The mean (SD) Injury Severity Score (ISS) was 8.1(8.6%). The overall case-fatality proportion was 3.8% and 75% of all the mortalities related to road traffic accidents.

**Conclusions:**

The main priorities in reducing the burden of injuries include: the young, male target group, improving pre-hospital and ambulance services, preventing road traffic accidents, improving road safety and the safety of motorcyclists (compulsory helmet use, safer vehicles, dedicated motorcycle lanes).

## Introduction

Nowadays, injuries are a serious health and socioeconomic issue around the world.^1-3^ They are the leading cause of death during the first four decades of life. ^4,5^ Injuries are believed to contribute to 10% of mortality globally. ^6^ With Low and Middle Income Countries (LIMCs) as the location of about 90% of injury mortality. ^7^ Each year more than 5 million people lose their life across the world due to some kind of injury. ^8-10^With current trends, it is assumed that the global burden of injuries will increase in the coming decades, especially in LMICs.^11^ Injuries are the second leading cause of mortality in Iran.^12^

In a 1966 White Paper, they were referred to as “the neglected disease of modern society” as there are fewer studies on injuries than on other diseases. This definition may describe the status of injuries in those LMICs which invest inadequate funds in public health programs as well as in injury research.^13-15^ An understanding of injury epidemiology makes it possible to develop appropriate approaches for injury prevention.^16^

Different studies have been published on the epidemiology of injuries in Iran in recent years.^17-19^ They have studied the epidemiology of injuries on a local level using only small sample sizes. Therefore, they cannot provide clear and beneficial information for decision-making on a macro level. Systematically reviewing the results and conclusions of these studies can provide useful information for decision-making and future research. Therefore, the aim of this study was to systematically review the epidemiology of injuries in Iran.

## Methods

This systematic review and meta-analysis study was conducted in 2016, using an approach adopted from the book “A Systematic Review to Support Evidence-Based Medicine”. ^20^

**Eligibility criteria**

The inclusion criteria for the study were: cross-sectional studies on the injuries, articles written in Iran, articles published in Persian and English, articles published from 1 January 2000 to 30 March 2016. Studies that focused on injuries in specific age groups of patients (children, elderly, etc.) or gender (male or female), studies that focused on specific kinds of injuries (road traffic accidents, burns, falls, etc.), community-based studies, conference presentations, case reports, interventional and qualitative studies were excluded from the study.

**Information sources**

Required data were collected searching for the following key words and their Persian equivalents; trauma, injury, accident, epidemiology, prevalence, pattern, etiology, risk factors and Iran. The following databases were searched: Google Scholar, PubMed, Scopus, MagIran, Iranian scientific information database (SID) and IranMedex. Some of the relevant journals and web sites were searched manually. The list of references for the selected articles was also reviewed. In the final stage of the literature review, we searched the gray literature and consulted experts. 

**Review process**

In the first phase of the review process, an extraction table was designed in which the following items were inserted: first author’s name, year of publication, city, data collection (number of hospitals and data collection time period), sample size, age (Mean ± SD), gender, major mechanisms of injuries, Road Traffic Accidents (RTAs) pattern, scene of injury, ambulance transportation, anatomical sites of injury, Injury Severity Score (ISS), mortality, and mortality due to RTAs. Validity of the data extraction table was improved by experts. A pilot study was conducted for further improvement of the extraction table. Two authors (SH and A-AS) had enough experience and knowledge in this field to be independently put in charge of the data extraction. 

In the first phase of the article selection procedure, articles with non-relevant titles were excluded. In the second phase, the abstract and the full text of articles were reviewed to select the articles that matched the inclusion criteria. Reference management (Endnote X5) software was used in order to organize and assess the titles and abstracts, as well as to identify duplicate entries. 

**Quality assessment**

Two reviewers (SH and A-AS) evaluated the reporting quality of articles according to the checklist of Strengthening the Reporting of Observational Studies in Epidemiology (STROBE) (Appendix 1).^21^ Those cases in which there was a disagreement between these reviewers were referred to a third author.

**Appendix 1:** STROBE Statement-checklist of items that should be included in reports of observational studies.

	Item No	Recommendation
**Title and abstract**	1	(a) Indicate the study’s design with a commonly used term in the title or the abstract (b) Provide in the abstract an informative and balanced summary of what was done and what was found
**Introduction**		
Background/rationale	2	Explain the scientific background and rationale for the investigation being reported
Objectives	3	State specific objectives, including any prespecified hypotheses
**Methods**		
Study design	4	Present key elements of study design early in the paper
Setting	5	Describe the setting, locations, and relevant dates, including periods of recruitment, exposure, follow-up, and data collection
Participants	6	(a) Cohort study—Give the eligibility criteria, and the sources and methods of selection of participants. Describe methods of follow-up Case-control study—Give the eligibility criteria, and the sources and methods of case ascertainment and control selection. Give the rationale for the choice of cases and controls Cross-sectional study—Give the eligibility criteria, and the sources and methods of selection of participants (b)Cohort study—For matched studies, give matching criteria and number of exposed and unexposed Case-control study—For matched studies, give matching criteria and the number of controls per case
Variables	7	Clearly define all outcomes, exposures, predictors, potential confounders, and effect modifiers. Give diagnostic criteria, if applicable
Data sources/ meas-urement	8*	For each variable of interest, give sources of data and details of methods of assessment (measurement). Describe comparability of assessment methods if there is more than one group
Bias	9	Describe any efforts to address potential sources of bias
Study size	10	Explain how the study size was arrived at
Quantitative variables	11	Explain how quantitative variables were handled in the analyses. If applicable, describe which groupings were chosen and why
Statistical methods	12	(a) Describe all statistical methods, including those used to control for confounding (b) Describe any methods used to examine subgroups and interactions (c) Explain how missing data were addressed (d) Cohort study—If applicable, explain how loss to follow-up was addressed Case-control study—If applicable, explain how matching of cases and controls was ad-dressed Cross-sectional study—If applicable, describe analytical methods taking account of sampling strategy (e) Describe any sensitivity analyses
**Result**		
Participants	13*	(a) Report numbers of individuals at each stage of study-eg numbers potentially eligible, examined for eligibility, confirmed eligible, included in the study, completing follow-up, and analyzed (b) Give reasons for non-participation at each stage (c) Consider use of a flow diagram
Descriptive data	14*	(a) Give characteristics of study participants (eg demographic, clinical, social) and infor-mation on exposures and potential confounders (b) Indicate number of participants with missing data for each variable of interest (c) Cohort study—Summarize follow-up time (eg, average and total amount)
Outcome data	15*	Cohort study Report numbers of outcome events or summary measures over time Case-control study—Report numbers in each exposure category, or summary measures of exposure
Main results	16	Cross-sectional study Report numbers of outcome events or summary measures (a) Give unadjusted estimates and, if applicable, confounder-adjusted estimates and their precision (eg, 95% confidence interval). Make clear which confounders were adjusted for and why they were included (b) Report category boundaries when continuous variables were categorized (c) If relevant, consider translating estimates of relative risk into absolute risk for a meaningful time period
Other analyses	17	Report other analyses done-eg analyses of subgroups and interactions, and sensitivity analyses
**Discussion**		
Key results	18	Summarize key results with reference to study objectives
Limitations	19	Discuss limitations of the study, taking into account sources of potential bias or imprecision. Discuss both direction and magnitude of any potential bias
Interpretation	20	Give a cautious overall interpretation of results considering objectives, limitations, multiplicity of analyses, results from similar studies, and other relevant evidence
Generalizability	21	Discuss the generalizability (external validity) of the study results
**Other information**		
Funding	22	Give the source of funding and the role of the funders for the present study and, if applicable, for the original study on which the present article is based

**Data analysis**

In the current study, due to the high heterogeneity in the report of studies’ results and some methodological issues, we could not conduct a quantitative analysis (Meta-Analysis methods). Data were analyzed manually. Descriptive statistics such as frequency, percentage, Mean ±Standard Deviation were used to report the results. Excel 2010 software was used to draw the graphs. 

## Results

**Fig.1:**
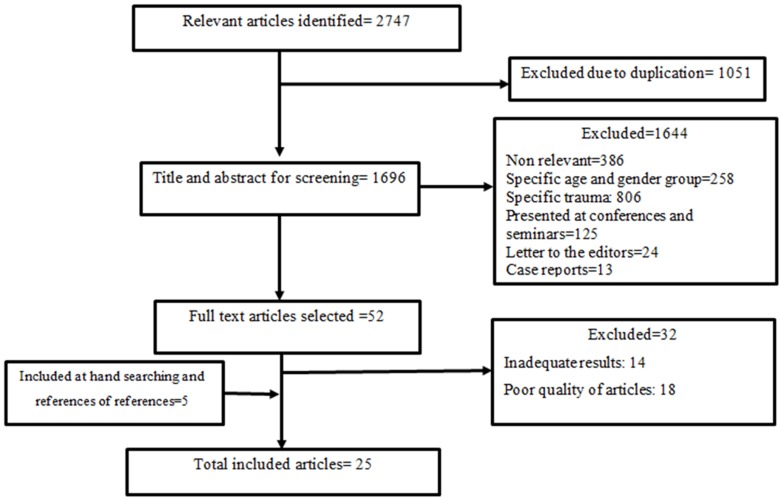
Bibliographical searches and inclusion process for injury epidemiology articles in Iran.

In this study, out of 2747 articles, 25 articles were finally found to be completely related to the study's objective and thus included in the study(Figure 1).

The results of the extracted data from the entered articles are summarized in Table 1.

**Table 1:** Characteristics of the included injury epidemiology articles in Iran

Author, Year	City/ district	Data Collection	Sample Size	Age (Mean ± SD)	Male (%)	Major Mechanisms Of Injuries	RTA* (%) Pattern	Place Of Injury (%)	Ambulance Transporta-tion (%)	Anatomical Sites Of Injury (%)	Injury Severity **(%)	Case fatali-ty propor-tion (%)	proportion of Mortality Due To RTA
Moini et al. 2000^73^	Tehran	3 hospitals (12 Months)	2662	28 ± 19	78	RTAs (37.8), Fall (29.7), and Blunt Objects (16.3)	Pedestrian (54.1), Motor-cycle (26.5), Car(16.8), and others (2.4)	-	14	-	Mild=65, Moderate=25, Severe=10, Mean ± SD=6.1 ± 5.5	2	-
Modaghegh et al. 2013^74^	Mashhad	One hospital (12 Months)	1544	30.1	54	RTAs (67.2), Fall(20.2), Blunt Objects (5.4), and others (7.4)	Pedestrians (40.1), Motorcyclists (33.2), and Car (25.6)	-	39.5	Head And Neck (42.5) and Lower Extremities (45.5)	Mild=47.3, Moderate=31.8, Severe=20.9, and Mean ± SD=10.3 ± 12.83	6.1	87.7
Fazel, et al. 2012 ^75^	Kashan	One hospital (60 Months)	22564	33.1± 10.9	75	RTAs (60.4)	-	Road(60.4),Workplace(15.1), Home(19.7), Sports (1.4), and others(3.4)	40	Head, Neck, Spine (31.2), Extremities (58.8), Ear, Nose, Throat (7.2), and others (2.8)	-	1.1	76
Sheikhghomi, et al.2015^76^	Tehran	One hospital (12 Months)	73	40.1 ± 20.3	67.1	Falls (47.9),RTAs (40.8), and others(11.3)	-	-	-	Head (23.8), Elbow and Forearm (19), Hip and Thigh(15.9), and Multiple Body Regions (14.3), and others(13.3)	Mean ± SD=7.26±7	3.7	-
Karami Joushin, et al. 2013^77^	Qom	- (12 Months)	29426	29 ±14.5	70	Strike (65.3), Fall (12.3), RTAs (11.1), and Others (11.2)	Motorcycle (53), Car (33), and Pedestrian (14)	Road(65), Workplace(8), Home(20), Sports Place (1), and others(6)	-	-	-	-	-
Rasouli, et al. 2011 ^78^	Tehran	One hospital (36 Months)	2991624	26.5±16.9	72.7	RTAs (31.9), Violence (25.5), Fall (10.9), and others (31.6)	Motorcycle (43.4), Car (37.5), and Pedestrian (19)	-	-	-	-	0.6	55.4
Karbakhsh, et al. 2009^79^	Kermanshah	One hospital(4 Months)	779	34.7±19.9	78.6	RTA (53.5),Fall (28.8)(10.1), and others (7.6)	Pedestrian (44.1), Car (29.7), Motorcycle (23.9), and Others (2.1)	Road(61.9), Workplace(4.9), Home(27.1), and others(6.2)	31.6	Head (27.6), Knee and Lower Leg (14.1), Abdomen, Lower Back, Lumbar Spine and Pelvis (17.8), Hip and Thigh(16.9), and others (23.6)	Mild=48.3, Moderate=33.6, Severe=18.1	7.8	68.8
Abbasi, et al. 2013^80^	Shiraz	One hospital (13 Months)	1217	26.6 ± 15.1	75.8	RTA (53.9),Fall (18.5), Violence (14.7), and others (12.9)	Car (42.9), Motorcycle(41.1), and Pedestrian (16.3)	-	-	Head and Neck (86.8), Face (18.4), Chest (24.2), Abdo-men (20.3), and Extremity and External Injuries (40.9)	1-7= 65.9, 8-16=27, 17-25=3.5, 25<=3.5		
Hossein, et al. 2009 ^81^	Rasht	One hospital (9 Months)	3598	31.85 ± 17.76	77.7	RTA (73.8),Fall (15.5), Violence (5.4), and others (5.2)	Motorcycle(47), Car (24), and Pedestrian (20), and others(9)	-	47	Head and Neck(82.4), Limb&Pelvic (37.7), Face (13.9),Spine(7), Chest(3.8), and Abdomen (3)		2.7	79
Sadeghi-Bazargani, et al. 2013^19^	Tabriz	One hospital (12 Months)	19530	31± 19.9	76.7	RTA Falls	-	-	-	-	-	.2	-
Shahrokh Yousefzadeh, et al. 2009^82^	Rasht	One hospital (6 Months)	1141	344± 18	78.2	RTAs (74.4),Fall (14.9), Violence (4.6), and others (6.1)	Motorcycle(47), and Pedestrian (23.1), Car (21.4), and others(8.5)	-	49.7	Head and Neck(80.8), Limb&Pelvic (25.4),Face (17.8),Spine(6.9), Chest(5.2), and Abdomen (4.3)	Mild=77.8, Moderate=11, Severe=11.4	5.17	83
Farzan-dipour, et al. 2007^83^	Kashan	One hospital (12 Months)	6415	27.7 ± 17.1	77.8	RTAs (47.5), Fall (29.9), Blunt Objects (9.3), Vio-lence (3.7), and others (9.6)	Motorcycle(60.6), Car (18.6), and Pedestrian (13.5), and others(7.3)	-	-	Hand(36.4), Head and Neck(28.4), Limb&Pelvic (28.3),Spine(2.2), Chest(1), and Abdomen (3.6)	-	-	78.1
Beigzadeh, et al. 2015^84^	Kerman	One hospital (12 Months)	10161	-	76.8	RTAs (49.7), Vio-lence (16.9),Fall (15.1), Occupa-tion (11.6), and others (6.7)	-	-	-	Limb&Pelvic (44.6), Head and Neck(18.8), Abdomen (18.1)Spine(12.2), and Chest(6.4),	-	-	-
Solhi, et al. 2010^85^	Arak	One hospital (12 Months)	813	-	74	RTAs (43), Occupa-tion (21), and others(36)	-	-	15	-	-	-	-
Khatami, et al. 2003^86^	Tehran	One hospital (12 Months)	1393	28.8 ± 17.3	89.3	RTAs (37), Fall (35), Cut (11.5), and others (16.5)	Motorcycle(42.1), Car (28.1), and Pedestrian (25.6), and others(4.2)	Road(43.7), Workplace(26), Home(17.5), and others(12.8)	35.1	-	Mild=75.6, Moderate=18.2, Severe=6.2	1	-
Salimi, et al. 2008 ^87^	Ahvaz	One hospital (7 Months)	1141	26.7 ± 17	83.4	RTAs (59), Fall (21), Blunt Objects (12.3), Cut (4.7), and others (3)	Pedestrian (35.9), Motor-cycle(35.3), Car (26.4), and others(2.4)	-	11.8	-	-	8.4	74
Khosravi, and Ebrahimi 2008 ^88^	Shahrood	One hospital (18 Months)	220	32.3 ± 18.6	79.1	RTAs (80), Fall (12.3), Violence (2.7), and others (2.3)	-	-	-	Limb&Pelvic (78.2), Head and Neck(71.4), Face (50.9), Chest(35), and Abdomen (29.1)	-	21.3	-
Amani, et al. 2009 ^89^	Ardabil	One hospital (6 Months)	955	28.7±18.7	69.9	Fall (38.5), Cut (22.1),RTAs (10.8), Violence (5.6), Burn (5), and others (18.1)	-	-	9	Limb&Pelvic (68.7), Head (24.1), Face (13.9), Chest(4.6), and Abdomen (2.6)	-	0.1	-
Zamani, et al. 2014^90^	Isfahan	3 hospitals (3 Months)	1363	30.5 ±17.35	73.6	RTAs (62.5), Fall (17.3), Cut (6.8), Blunt Objects (5.7), and others (7.7)	Motorcycle(78.8), Pedes-trian (15.3), and Car (5.9)	-	49.6	Limb&Pelvic (44.9), Head and Neck (30.8), Face (13.9), Chest(3.3), Abdomen (4.3), and Spine(4.3),	-	0.7	-
Chardoli and Rahimi-Movaghar 2006^91^	Zahedan	One hospital (12 Months)	768	22.8 ±16.1	82	RTAs (59.4), Fall (18.1), and others (22.5)	-	-	-	-	-	8	
Soroush, et al. 2008^92^	Shiraz	2 hospitals (6 Months)	1765	33±20	81.3	RTAs (53.3), Fall (25.9), Cut (8.8), Blunt Objects (7.2), and others (9.8)	Motorcycle(42.3), Pedes-trian (29), Car (21.1), and others(7.6)	Road(61), Work-place(10.5), Home(27.1)	43	-	Mild=43.4, Moderate=34.2, Severe=22.4, Mean ± SD=10.2 ± 10.9	2	75
Moosazadeh, et al. 2013^93^	Mazandaran	15 hospitals (12 Months)	58750	29.9±17.01	71.7	RTAs (39.8), Fall (31.8), Violence (5.3), Burn (5.2), and others (17.9)	Car (44.1), Motorcy-cle(41.9), and Pedestrian (14)	Road(47.9), Workplace(10.6), Home(33.7), and others(6.8)	-	-	-	0.8	-
Fazel, et al. 2008^94^	Kashan	3 hospitals (6 Months)	18166	29.2±19.9	76.5	RTAs (50.5), Fall (32.3), Violence (6.2), and others (11)	Motorcycle(59), and Pedestrian (22), Car (13), and others(6)	-	-	-	-	1.3	83
Adib-Hajbaghery and Ma-ghaminejad 2014^95^	Kashan	One hospital (6 Months)	400	-	75.2	RTAs (87.2), Fall (9.2), Violence (2.5), and others (1.1)	Motorcycle(61.3), Car (23.8), and Pedestrian (14.9)	Road(94.2), Workplace(3.3), and Home(2.5)	-	Upper Limb (65.1), Head and Neck (60), Lower Limb (60.8), Chest(11.7), and Abdomen (32.2)	-	-	-
Zargar, et al. 2001^96^	Tehran	3 hospitals (12 Months)	58013	27±16	80	Blunt Objects (50), RTAs (19), Cut (18.9), Fall (7.5), and others (4.6)	Pedestrian (47), Motorcycle(30), Car (19), and others(4)	Road(34), Work-place(35), Home(30), and Sports(1)	22	Head and Neck (46), Extremities (43), and Limb & Pelvic (44.9)	Mild=92, Moderate=6, Severe=2, and Mean ± SD= 7.1±7.2	1	-

*RTA: Road Traffic Accidents ** Mild<7 Moderate7-12 Severe>12 ranging from 1 to 75

The pattern of injuries had been studied in 16 cities in 15 provinces. In 24 studies, where the number of studied hospitals had been indicated, data were collected from 47 hospitals over a 324 Months period. The total number of studied injury cases was 3 234 481 and the mean ±standard deviation of the age of the cases was 30±17.4. In all of the studies, the number of the males was higher than the females (75.75% vs. 24.3%). Only 31.3% of injured cases had been referred to hospitals by ambulance.

**Figure 2:**
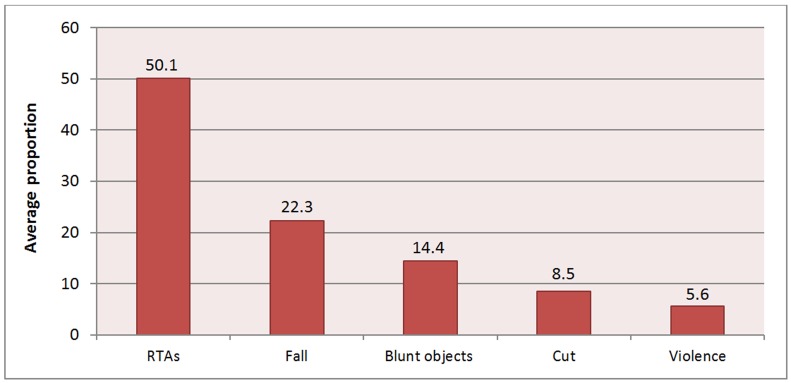
Average proportion of the mechanisms of injuries reported in various studies in Iran

RTAs, followed by fall accidents, were the leading cause of injuries in the vast majority of the studies (21 studies out of 25) (Figure 2).

**Figure 3:**
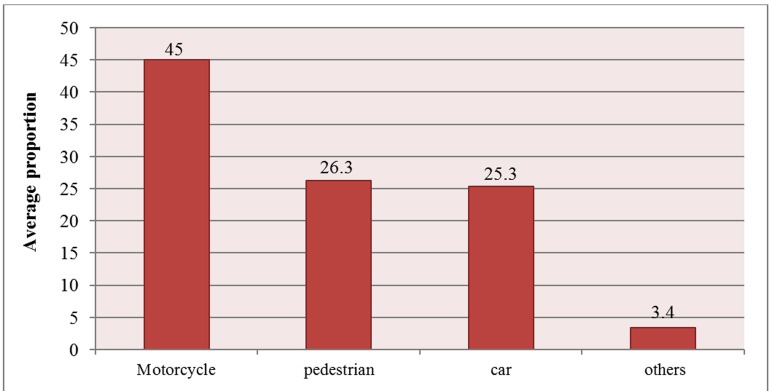
Average proportion of the different modes of road traffic injuries reported in various studies in Iran

Among RTAs injuries, the highest frequency was caused by motorcycle crashes (Figure 3)

**Figure 4:**
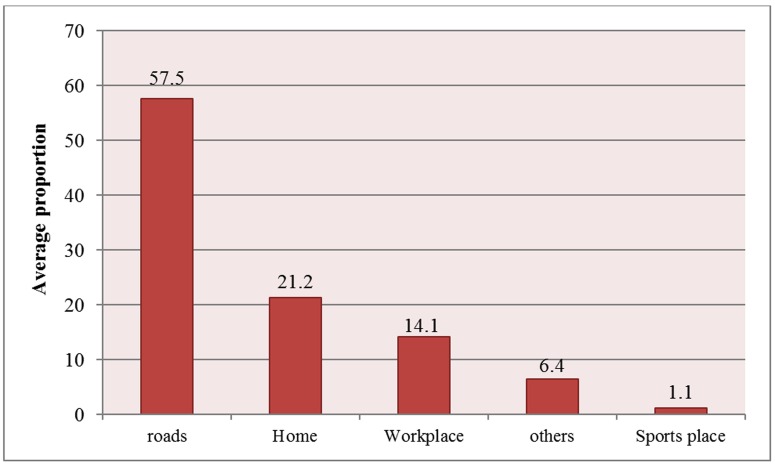
Average proportion of the place of injuries reported in various studies in Iran

The majority of injuries occurred on roads including urban roads, rural roads and highways (Figure 4).

**Figure 5:**
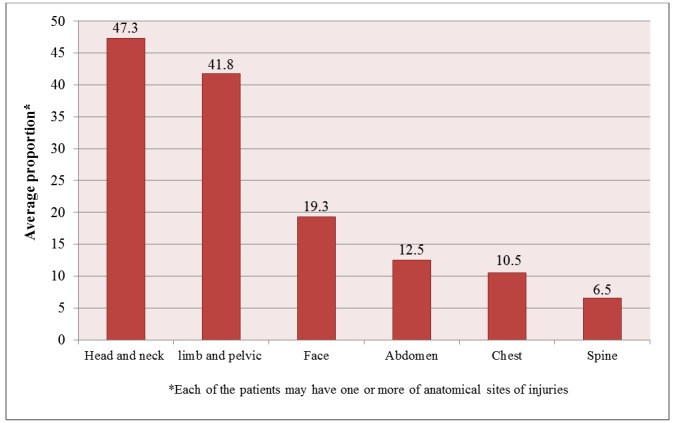
Average proportion of the anatomical sites of injuries reported in various studies in Iran

Head and neck injuries were the most common during accidents (47.3% of cases) while the spinal cord suffered the least number of injuries (6.5% of cases)(Figure 5).

The mean ± standard deviation of the severity of injuries was calculated to be 8.1±8.6 (average proportion of mild injuries was 64, moderate injuries 23 and severe injuries 13).

In these studies, the case-fatality proportion ranged from 0.1 to 21.3 with the total mean of 3.8. RTAs accounted for 75% of injury-caused case-fatality proportion.

## Discussion

According to this study, young people account for a large number of injury victims which is consistent with the majority of studies conducted in this area.^22-25^ Since the studied cases were often of working and productive age and many of them were the breadwinners of their families, their injuries impose serious socioeconomic costs in comparison with other groups. Therefore, it is important to pay more attention to the prevention of injuries in this group. In addition, the frequency of injuries is higher in males than the females. This is consistent with the majority of studies conducted in other countries.^26-29^ The studies that have been conducted in this area have pointed out several factors which may be applicable to Iran. Similar to their counterparts in most LMICs compared with High Income Countries (HICs), Iranian males are more vulnerable to injury risks due to the special socio-cultural context of Iran (more driving, more occupational threats, violence-induced injuries and so on). Nevertheless, some types of injuries such as fractures and injuries that occur at home may be seen more in females than males due to females’ gender roles or the nature of their duties.^30,31^

According to the reviewed studies, less than one third of the injured cases were referred to hospitals by ambulance. This is a poor performance compared with other countries.^32,33^ The study by Naghavi and colleagues in 12 provinces of Iran in 2002 showed that 7.2% and 90.8% of injury cases were referred to hospitals by ambulance and conventional vehicles, respectively. ^12^ Whereas according to the results of Zafarghandi and Moeinian, only 5% of injury cases in Tehran had been referred to hospitals by ambulance in 1999. ^34^ This may indicate the improved status of pre-hospital care and the quantitative and qualitative promotion of the ambulances service in Iran in recent years. Given the importance of the rapid and professional transfer of the injured to hospitals, this condition cannot be considered as satisfactory since it is far below the standard. Thus, additional plans and endeavors for the qualitative and quantitative promotion of pre-hospital services seem necessary. 

According to the current study’s result, RTAs account for the majority of injuries which is consistent with the results of most of studies in this field. ^35-40^ From 2005 to 2008, Iran had the world’s highest road injury death rate. ^41^ At that time, the study of Khorasani-zavare and colleagues showed that there are many obstacles to the prevention of RTAs. ^42^ Since that time, the Iranian authorities have designed and implemented many interventions in order to prevent RTAs ^43-47^ so that ,according to a WHO 2015 report, RTAs mortality has shown a descending trend decreasing from 40 cases in every 100 thousand cases in 2005 to 24 cases in every 100 thousand cases in 2014.^48^ Despite the advances in the prevention of RTAs in recent years, they continue to be the primary cause of injuries and the second greatest cause of mortality in Iran.^49-51^ It seems that there still are many barriers to preventing RTAs in Iran. In this regard more valid studies are needed in this field. 

Based on the results, the rate of motorcycle accidents and their injuries is higher among RTAs cases which is consistent with the numbers obtained in other studies.^52-55^ Crash injuries and mortality among motorcyclists have been reduced in HICs communities to a large extent, thanks to the promoted preventive actions, improved injury diagnosis and treatment and enhanced injury centers and care system. ^56^ Many efforts have been made in Iran in recent years in order to reduce the crash rate among motorcyclists. Harsher punishment for a motorcyclist who breaks the law is a special plan that has been considered by the traffic department. The study conducted by Yunesian and colleagues revealed that the daily mean of the number of traffic-caused injured cases referred to Sina hospital during the first month of the implementation of this plan shows an ascending trend compared with the previous month as well as with the corresponding month of last year. However, the occurrence of severe traffic-caused injuries as well as severe head and neck injuries have decreased. ^57^ Given the fact that motorcycle crashes are still the main cause of injuries in Iran and other LMICs, ^58^ considerable amount of attention should be paid to this field. A set of plans for promoting helmet use by motorcyclists and designing special lanes for them may yield better results.^59-61^

The results of these studies showed that the vast majority of accidents have occurred on roads. Since RTAs are the main cause of the injuries, roads are of course the primary accident scene. According to a 2009 WHO report, roads in Iran are sub-standard from a safety point of view.^62^ In addition, the results of Khorasani-zavare and colleagues (2009) confirm that the poor road safety in Iran is the main obstacle to accident prevention. ^42^ The results of Jafari and colleagues also revealed the poor safety standard form 2001 to 2005. ^63^ Nevertheless, according to a 2013 WHO report, the safety of Iran's roads has improved slightly in the past few years. ^64^ But more initiatives are needed in order to achieve a satisfactory level. 

Head and neck injuries are the most common. This conclusion is consistent with a lot of studies conducted outside Iran.^65-68^ It seems that RTAs are the main cause of these injuries. Therefore, safety considerations should be particularly taken into account regarding vehicles and motorcycles. Installing standard air bags, and making seat belt and helmet use compulsory are among the most important courses of action. 

The total mean of case-fatality proportion was computed to be 3.8 where RTAs account for 75% of this number. Although this appears a negligible number, its severity will be highlighted by paying attention to two important facts: 1) only those cases that were referred to hospitals were included in this number. It should be noted that more cases die at the accident scene and they are not referred to hospitals. Thus, injury case-fatality proportion is definitely higher than 3.8. 2) Since about 4 deaths happen in every 100 thousand cases referred to hospitals, the case-fatality proportion will be a high number considering thousands of people who are referred daily to hospitals due to injuries resulting from accidents. Problems associated with recording systems and underestimation of problems can be added to this challenge. This study observed no special increasing/decreasing trend of injury case-fatality proportion.^69-72^ Observing safety considerations at different locations including roads, work places, and homes and so on, improving pre-hospital care systems and capabilities, improving care systems and methods for injury cases, public training courses and different preventive programs can reduce injury-related mortality. 

Despite serious follow ups, some reports and theses were not available. This was an important limitation of this study. Non-homogenous results were another limitation of this study making it impossible to conduct meta-analyses and Standardized Mean Difference (SMD). In this regard this limitation should be considered when interpreting the results of this study. 

## Conclusion

According to the results obtained in the current study, it is recommended that managers and policy-makers pay more attention and give priority to the following items: paying more attention to the prevention of accident injuries among men especially young men, qualitative and quantitative promotion of pre-hospital services and ambulance services, promoting the safety of roads and vehicles, developing a set of actions for promoting the safety of motorcycles (manufacturing safe motorcycles, creating special motorcycle lanes, and laws to make helmet use compulsory) and improving emergency care, especially for head and neck injuries. 
